# Perineural Invasion in Early-Stage Cervical Cancer: Marker of Aggressive Pathology and Increased Recurrence Risk

**DOI:** 10.3390/biomedicines14030591

**Published:** 2026-03-05

**Authors:** Lihua Tan, Hongyao Li, Tianyi Liu, Wei Mao, Yan Song, Dan Zhao

**Affiliations:** 1 Department of Gynecologic Oncology, National Cancer Center/National Clinical Research Center for Cancer/Cancer Hospital, Chinese Academy of Medical Sciences and Peking Union Medical College, Beijing 100021, China; b2024003112@student.pumc.edu.cn (L.T.); s2023003019@student.pumc.edu.cn (H.L.); maowei_sdu@126.com (W.M.); 2 Department of Medical Oncology, National Cancer Center/National Clinical Research Center for Cancer/Cancer Hospital, Chinese Academy of Medical Sciences and Peking Union Medical College, Beijing 100021, China; liutianyi2111@163.com; 3 Department of Pathology, National Cancer Center/National Clinical Research Center for Cancer/Cancer Hospital, Chinese Academy of Medical Sciences and Peking Union Medical College, Beijing 100021, China

**Keywords:** cervical cancer, perineural invasion, early stage, risk stratification, recurrence, prognosis, TCGA, transcriptomics, immune microenvironment, microRNA

## Abstract

**Background:** Perineural invasion (PNI) is associated with aggressive tumor behavior in several malignancies, but its independent prognostic value in early-stage cervical cancer remains uncertain. We evaluated the clinical significance of PNI and explored molecular and immune features associated with PNI. **Methods:** We retrospectively analyzed 499 patients with FIGO 2009 stage IB–IIA cervical cancer treated with radical hysterectomy and pelvic lymphadenectomy. Associations between PNI, clinicopathological variables, recurrence-free survival, and overall survival were assessed using Kaplan–Meier methods and Cox regression. An independent cohort of 286 cervical cancers from The Cancer Genome Atlas (TCGA) was analyzed to characterize PNI-associated transcriptomic patterns, pathway enrichment, immune cell composition, and microRNA profiles. **Results:** PNI was identified in 11.6% of cases and was associated with larger tumor size, deep stromal invasion, and lymphovascular space invasion. PNI was not an independent prognostic factor in the overall cohort; however, it was associated with increased recurrence risk in the subgroup without high-risk factors and not meeting Sedlis criteria, with a modest improvement in 5-year recurrence discrimination when incorporated into Sedlis-based models. In TCGA, PNI was associated with differential gene expression and enrichment of oncogenic and immune-related pathways, an increased estimated abundance of resting mast cells, and six differentially expressed microRNAs. **Conclusions:** In early-stage cervical cancer, PNI is strongly correlated with established adverse pathological features and shows a subgroup-specific association with recurrence in an otherwise low-risk postoperative population. The multi-omics findings are exploratory and support biological hypotheses regarding tumor–nerve–immune interactions; external validation is needed before PNI can be used to guide postoperative management.

## 1. Introduction

Cervical cancer is the fourth most commonly diagnosed cancer among women worldwide, accounting for approximately 7.7% of female cancer-related deaths [1]. For early-stage disease, radical hysterectomy (RH) with pelvic lymphadenectomy remains the standard treatment, except for patients requiring fertility preservation. Postoperative risk stratification guides adjuvant therapy decisions, with radiotherapy recommended for patients presenting pathological high-risk factors such as lymph node metastasis, parametrial invasion, or positive surgical margins [2].

Perineural invasion (PNI), first described by Cruveilhier in 1835, is defined as tumor cell infiltration into the epineurium, perineurium, or endoneurium, or encirclement of at least one-third of the nerve’s circumference [3]. PNI is recognized as a distinct metastatic route, alongside lymphatic, hematogenous, direct invasion, and transcoelomic spread [4], and has been reported across a variety of solid tumors, including those of the pancreas, prostate, head and neck, stomach, biliary tract, colorectum, and breast. PNI is generally associated with aggressive tumor behavior, worse progression-free survival, and poorer overall survival [4,5,6,7,8]. In cervical cancer, PNI was first documented in 2003. A meta-analysis including seven retrospective cohort studies comprising 1561 patients with stage IA–IIB disease reported an overall PNI incidence of 12.1% (95% CI: 8.0–18.4%; I^2^ = 96%) [9]. PNI was significantly associated with adverse pathological features, including advanced clinical stage, larger tumor size, lymph node metastasis, deep stromal invasion, lymphovascular invasion, positive surgical margins, and parametrial involvement. Although PNI has been linked to unfavorable pathological characteristics and increased recurrence risk, its independent prognostic value remains controversial [10,11,12,13]. A recent study further suggested that PNI may be more appropriately classified as an intermediate-risk factor rather than a high-risk feature [14].

Mechanistically, PNI involves dynamic interactions between tumor cells, nerve fibers, and the surrounding microenvironment [15,16]. Neurotrophic factors (e.g., NGF/TrkA, BDNF/TrkB), chemokine signaling (e.g., CCL2–CCR2 axis), and matrix metalloproteinases (e.g., MMP2, MMP9, MMP12) facilitate nerve-directed tumor migration and extracellular matrix remodeling [15,16,17,18,19]. Additionally, adhesion molecules such as MUC1, MUC4, and L1CAM [20,21,22,23], and neuropeptides including PACAP and NMB, promote PNI by enhancing cell adhesion and Schwann cell activation [24,25]. Recent evidence also implicates immune cell infiltration and microenvironmental remodeling in the development of PNI. Notably, integrative genomic analyses have identified alterations such as FBXW7 mutations and a three-gene expression signature (MT1G, NPAS1, and SPRY1) associated with PNI, suggesting links to MYC activation and an immunosuppressive tumor microenvironment; however, the molecular features of PNI in cervical cancer require further investigation [26].

This study aims to evaluate the prognostic significance of PNI and its potential role in informing postoperative treatment strategies in early-stage cervical cancer. Furthermore, we investigated the genomic and immunological characteristics associated with PNI using data from The Cancer Genome Atlas (TCGA) cohort.

## 2. Materials and Methods

### 2.1. Study Design and Patient Cohorts

This study included two independent patient cohorts: (i) NCC-RH cohort: This cohort included 499 patients with stage IB–IIA (FIGO 2009) cervical cancer, including squamous cell carcinoma (SCC), adenocarcinoma (AC), and adenosquamous carcinoma (ASC), who underwent radical hysterectomy with pelvic lymphadenectomy at the Cancer Hospital, Chinese Academy of Medical Sciences, from January 2017 to December 2019. Para-aortic lymphadenectomy was performed in selected cases. Patients with distant metastasis, other malignancies, or incomplete pathological or follow-up data were excluded. All pathological diagnoses were reviewed by gynecologic pathologists. (ii) TCGA cohort: A total of 286 cervical cancer cases from The Cancer Genome Atlas (TCGA) were included after confirming PNI status via histopathological review of whole-slide hematoxylin and eosin (H&E) images by experienced pathologists.

### 2.2. PNI Assessment

PNI was diagnosed based on hematoxylin and eosin (H&E)-stained 4 μm paraffin-embedded sections. All H&E-stained sections from each patient were reviewed to assess PNI. Only definite tumor invasion into or through the nerve sheath was considered PNI, whereas mere proximity or contact between tumor cells and nerves was not classified as PNI. The final diagnosis and reporting were established based on independent evaluation by two pathologists. When identification was uncertain, S100 immunohistochemistry was used to assist in the diagnosis.

For the TCGA cohort, H&E-stained histopathology images and corresponding genomic, phenotypic, and survival data were downloaded from public TCGA repositories (https://portal.gdc.cancer.gov/analysis_page?app=Downloads, accessed on 6 June 2024). After quality control, 286 cervical squamous cell carcinoma (CESC) cases with available PNI status were included for downstream analyses.

### 2.3. Molecular Analysis

Differential gene expression analysis was performed using the limma package (v3.42.2), with thresholds set at absolute log2-fold change (FC) > 1 and a false discovery rate (FDR) < 0.05. Identified differentially expressed genes (DEGs) were subjected to Gene Ontology (GO) and Kyoto Encyclopedia of Genes and Genomes (KEGG) pathway enrichment using the clusterProfiler package (v3.14.3), with gene annotation provided by org.Hs.eg.db (v3.10.0).

The tumor immune microenvironment was evaluated using two computational algorithms. CIBERSORT was applied to estimate the relative abundance of 22 immune cell types, while ESTIMATE was used to calculate stromal score, immune score, ESTIMATE score, and tumor purity. Prognostic analysis of PNI-associated microRNAs (miRNAs) was conducted by stratifying patients according to expression levels and comparing survival outcomes using Kaplan–Meier curves.

Data visualization was performed using ggplot2 (v3.3.2), gplots (v3.1.0), and GOplot (v1.0.2). All analyses were conducted in R version 4.3.1.

### 2.4. Statistical Analysis

Chi-square tests were used to compare clinicopathological characteristics between groups. Univariate and multivariate logistic regression analyses were conducted to identify factors associated with recurrence. Kaplan–Meier survival analysis was performed to assess recurrence-free survival (RFS) and overall survival (OS), with differences evaluated using the log-rank test. Cox proportional-hazards models were used to identify independent prognostic factors.

To evaluate the predictive value of PNI, a 5-year recurrence prediction model was constructed using logistic regression. Model performance was assessed by receiver operating characteristic (ROC) curve analysis, and the area under the curve (AUC) was calculated with corresponding 95% confidence intervals. A two-sided *p*-value < 0.05 was considered statistically significant. All statistical analyses were performed using IBM SPSS Statistics version 25 and R version 4.3.1.

## 3. Results

### 3.1. Clinical and Pathological Characteristics

We included 499 cervical cancer patients (FIGO 2009 stage IB–IIA) who underwent radical hysterectomy with pelvic lymphadenectomy. The mean age was 47.6 years. Most patients (84.2%) had squamous carcinoma, while 15.8% had adenocarcinoma or adenosquamous carcinoma. Stage IB disease accounted for 83.8% of the cohort. PNI was confirmed in 58 patients (11.6%), and lymph node metastasis (LNM) in 65 patients (13.0%). A total of 43 patients (8.6%) experienced recurrence, with a median time to recurrence of 17 months (range: 5–59 months). During follow-up, 19 patients (3.8%) died from cervical cancer. Clinical and pathological data are summarized in Table 1.

PNI was significantly associated with larger tumor size (*p* = 0.002), vaginal invasion (*p* = 0.017), deeper stromal invasion (*p* < 0.001), and lymphovascular space invasion (LVSI) (*p* = 0.045), but not with age, FIGO stage, histological type, tumor grade, or LNM. No significant association was found between PNI and 5-year recurrence-free survival (RFS) or overall survival (OS) (*p* > 0.05).

### 3.2. Prognostic Factors for Survival

Univariate analysis (Table 2) showed that larger tumor size, deeper stromal invasion, LVSI, LNM, and meeting the Sedlis criteria were significantly associated with poorer 5-year RFS (*p* < 0.05). Tumor size, LVSI, LNM, and Sedlis high-risk classification were also associated with worse 5-year OS (*p* < 0.05).

Multivariate analysis (Table 3) identified tumor size as an independent risk factor for 5-year OS, while LVSI and LNM were independent predictors of 5-year RFS. PNI was not independently associated with RFS or OS.

### 3.3. Prognostic Value of PNI in Patients Not Meeting the Sedlis Criteria

**Figure 1:**
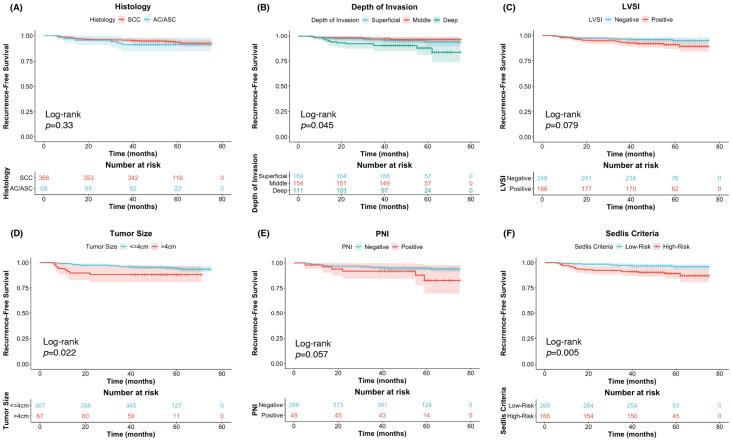
Recurrence-free survival of FIGO IB-IIA cervical cancer patients without lymph node metastasis after radical hysterectomy. (**A**) Kaplan–Meier survival curves for recurrence-free survival (RFS) in cervical cancer patients with FIGO stage IB-IIA treated with RH without LNM stratified by histology; (**B**) depth of invasion; (**C**) lymphovascular space invasion; (**D**) tumor size; (**E**) perineural invasion; (**F**) Sedlis criteria. SCC: Squamous Cell Carcinoma; AC: Adenocarcinoma; ASC: Adenosquamous Carcinoma; LVSI: Lymphovascular Invasion; PNI: Perineural Invasion.

Meeting the Sedlis criteria was defined as the presence of any one of the following risk factors: LVSI (+) with deep one-third stromal invasion; LVSI (+) with middle one-third stromal invasion and tumor size ≥2 cm; LVSI (+) with superficial one-third stromal invasion and tumor size ≥5 cm; or LVSI (–) with middle or deep one-third stromal invasion and tumor size ≥4 cm [2]. Among 434 patients with negative lymph nodes, negative parametrial invasion, and negative surgical margins, 268 (61.8%) did not meet Sedlis criteria. Within this subgroup, 19 patients (7.1%) had PNI, and 10 (3.7%) experienced recurrence within five years (range: 7–59 months). Kaplan–Meier curves for different prognostic factors in these patients are shown in Figure 1.

**Figure 2:**
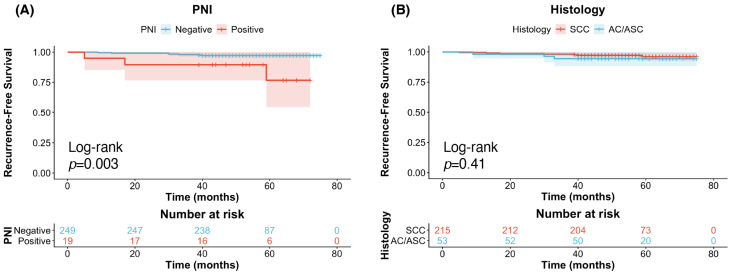
Recurrence-free survival in FIGO IB–IIA cervical cancer patients not meeting Sedlis criteria after radical hysterectomy. (**A**) Kaplan–Meier survival curves for recurrence-free survival (RFS) in cervical cancer patients with stage IB-IIA treated with RH who did not meet Sedlis criteria stratified by perineural invasion; (**B**) by histology. PNI: Perineural Invasion; SCC: Squamous Cell Carcinoma; AC: Adenocarcinoma; ASC: Adenosquamous Carcinoma.

Kaplan–Meier analysis revealed that among patients not meeting Sedlis criteria, those with PNI had significantly shorter RFS than those without PNI (*p* = 0.003), whereas no other pathological factors—including vaginal invasion, tumor differentiation, or histological type—were significantly associated with RFS in this subgroup (Figure 2). In contrast, among patients who did meet Sedlis criteria, PNI did not significantly impact RFS.

**Figure 3:**
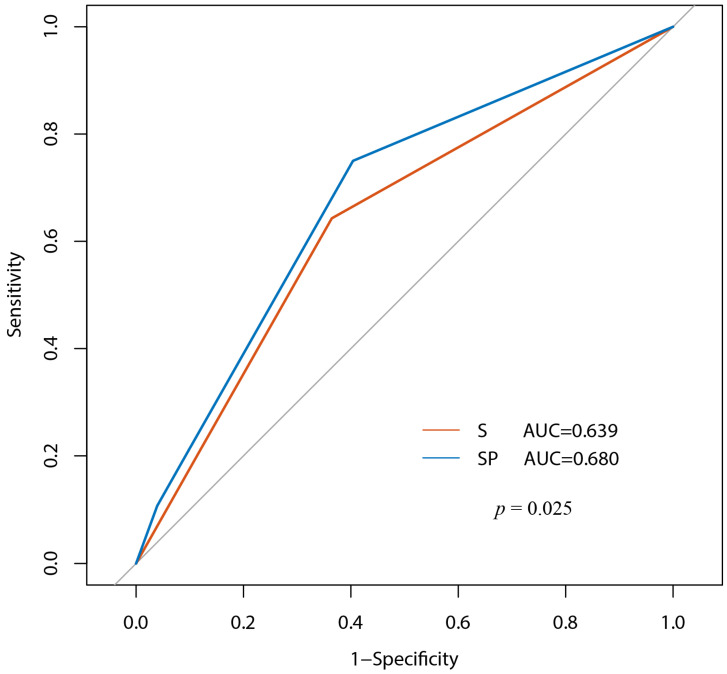
The receiver operating curves for cervical cancer recurrence in patients without lymph node metastasis. S: Sedlis criteria. SP: Sedlis criteria plus PNI.

Among patients not meeting the Sedlis criteria, adding PNI to the model significantly improved the prediction of 5-year recurrence, with the AUC increasing from 0.639 (Sedlis criteria alone) to 0.680 (Sedlis + PNI) (*p* < 0.05) (Figure 3).

### 3.4. Immune Microenvironment and Gene Expression

**Figure 4:**
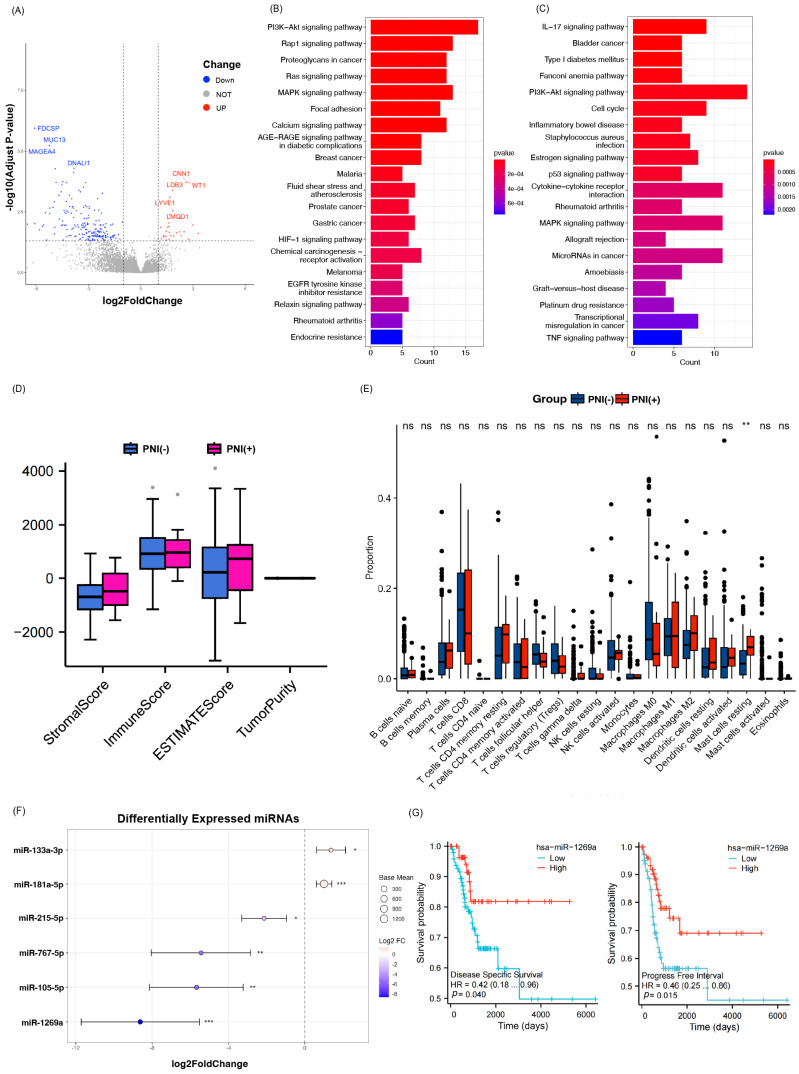
Molecular features associated with PNI in cervical cancer. Significance thresholds are indicated: no significance (ns), *p* < 0.05 (*), *p* < 0.01 (**), *p* < 0.001 (***). (**A**) Differential expression analysis between PNI-positive and PNI-negative CESC samples; (**B**) KEGG pathway enrichment analysis of upregulated DEGs in the PNI-positive group; (**C**) KEGG pathway enrichment analysis of downregulated DEGs in the PNI-positive group; (**D**) Comparison of stromal scores, immune scores, ESTIMATE scores, and tumor purity between PNI-positive and PNI-negative groups; (**E**) Bar plot showing the relative fractions of 22 immune cell types estimated by the CIBERSORT algorithm in PNI-positive and PNI-negative groups; (**F**) Differential expression of miRNAs between PNI-positive and PNI-negative CESC samples; (**G**) Prognostic value of hsa-miR-1269a expression in cervical cancer. PNI: Perineural Invasion.

To characterize the tumor immune microenvironment associated with PNI in CESC, we first applied the ESTIMATE algorithm. PNI-positive samples showed a trend toward higher immune and stromal scores and lower tumor purity compared with PNI-negative samples, although these differences did not reach statistical significance (Figure 4D). Using the CIBERSORT algorithm to estimate the proportions of 22 immune cell types, resting mast cells were identified as the only population with a significant difference, exhibiting a higher proportion in the PNI-positive group (*p* < 0.05; Figure 4E), suggesting a potential association with the perineural invasive phenotype.

We then examined transcriptional alterations associated with PNI status. Differential expression analysis using the limma package (v3.42.2) identified 229 differentially expressed genes (|log2FC| > 1, FDR < 0.05) between PNI-positive and PNI-negative samples, as illustrated in the volcano plot (Figure 4A). Functional enrichment analysis using clusterProfiler (v3.14.3) revealed significant enrichment of immune- and cancer-related pathways. Specifically, genes upregulated in the PNI-positive group were enriched in the PI3K/AKT and MAPK signaling pathways (Figure 4B), whereas downregulated genes were enriched in the p53 and IL-17 signaling pathways (Figure 4C), suggesting that PNI may be associated with dysregulated oncogenic and immune signaling in CESC.

### 3.5. PNI-Associated miRNAs

To identify microRNAs associated with PNI in cervical cancer, we performed differential expression analysis using RNA-seq data from PNI-positive and PNI-negative samples. Six miRNAs were found to be significantly differentially expressed (adjusted *p* < 0.05). Among them, miR-1269a, miR-105-5p, miR-767-5p, and miR-215-5p were significantly downregulated in the PNI-positive group, while miR-181a-5p and miR-133a-3p were upregulated.

A forest plot visualized the log2-fold changes and 95% confidence intervals (Figure 4F). Point size indicates average expression level, and color denotes the direction and magnitude of change. Significance thresholds are indicated: *p* < 0.05 (*), *p* < 0.01 (**), *p* < 0.001 (***). Notably, miR-1269a (log2FC = –8.62, adjusted *p* < 0.01) and miR-105-5p (log2FC = –5.68, adjusted *p* = 0.0013) showed the most pronounced downregulation. These miRNAs may contribute to regulatory networks underlying perineural invasion and warrant further functional investigation.

### 3.6. Prognostic Role of hsa-miR-1269a

To assess the prognostic relevance of hsa-miR-1269a, patients were stratified into high- and low-expression groups based on the median expression level, and Kaplan–Meier survival analysis was performed.

Patients with higher hsa-miR-1269a expression had significantly longer progression-free interval (PFI) (*p* = 0.0015) and improved disease-specific survival (DSS) (*p* = 0.04) (Figure 4G). These findings suggest that hsa-miR-1269a may act as a protective biomarker in cervical cancer, potentially modulating tumor aggressiveness, especially in the context of PNI.

## 4. Discussion

In our analysis, PNI was associated with increased recurrence primarily in the subgroup lacking other Sedlis-defined intermediate-risk factors. One possible explanation is that the prognostic contribution of PNI may be obscured when stronger risk factors, such as lymph node metastasis, positive margins, or parametrial involvement, are present; in the absence of these confounding factors, the prognostic relevance of PNI may become more apparent. These findings suggest that PNI may help identify higher-risk individuals within a traditionally low-risk population and could serve as a useful adjunct in future risk stratification models.

Consistent with previous reports, PNI was associated with several established prognostic factors but was not an independent predictor of recurrence in the overall cohort of early-stage cervical cancer patients. However, in patients who did not meet the Sedlis criteria, stratified analysis in our cohort identified PNI as an independent prognostic factor for recurrence. Given that this subgroup included 268 patients with only 10 recurrence events, the statistical power remains limited, and the stability of this finding should be interpreted with caution. Larger, multi-center cohorts are warranted to validate this observation. This observation extends prior research, as few studies have specifically evaluated PNI within Sedlis-defined subgroups. Together, these findings indicate that incorporating PNI status alongside the Sedlis criteria may improve recurrence risk assessment and assist clinical decision-making regarding adjuvant therapy.

Although previous literature has frequently reported associations between PNI and adverse pathological features, its prognostic relevance remains inconclusive. Memarzadeh et al. identified PNI as a predictor of recurrence, whereas Horn et al. found PNI to be independently associated with poorer overall survival but not recurrence. Conversely, Zhang et al. and Long et al. did not observe significant associations between PNI and clinical outcomes [10,11,12,13]. A study by Wan et al. reported that PNI-positive patients with a single intermediate-risk factor exhibited similar progression-free and overall survival to patients meeting Sedlis criteria, suggesting a potential role for PNI as an intermediate-risk factor [14]. However, the contribution of PNI to recurrence risk in early-stage disease remains poorly defined.

Our study provides a preliminary exploration of the molecular features associated with PNI in cervical cancer by integrating transcriptomic, immune, and miRNA-level analyses. PNI is recognized as an alternative route of tumor spread and has been associated with increased aggressiveness and unfavorable prognosis in multiple malignancies. However, its biological basis in cervical cancer remains incompletely understood. By characterizing transcriptional alterations, immune microenvironment features, and miRNA expression patterns linked to PNI, this study offers initial insights into potential mechanisms and generates hypotheses for further experimental validation.

CIBERSORT analysis showed that resting mast cells were significantly more abundant in PNI-positive tumors, suggesting a potential role in perineural invasion. Mast cells, derived from bone marrow progenitors and residing at host–environment interfaces, are key innate immune cells involved in allergic and inflammatory responses. Beyond their classical roles in IgE-mediated hypersensitivity and cytokine release, accumulating evidence indicates that mast cells can also play important roles in tumor biology. Through degranulation and the release of cytokines, chemokines, proteases, and growth factors, mast cells actively remodel the tumor microenvironment. They interact with cytotoxic T cells, NK cells, and stromal and tumor cells, thereby shaping immune contexture and potentially facilitating tumor invasion and metastasis [27]. In addition to their microenvironmental effects, mast cells serve as critical mediators of neuro-immune communication. They express receptors for neurotransmitters and neuropeptides and, in turn, release mediators such as histamine and tryptase that modulate neuronal excitability and phenotype, forming a bidirectional signaling network involved in inflammation, pain perception, and disease progression [28]. Consistent with this neuro-immune regulatory role, studies in breast cancer have reported a positive association between PNI and intratumoral mast cell density. Mast cells are known to promote lymphangiogenesis through secretion of VEGF-C and VEGF-D, suggesting that mast cell infiltration may facilitate PNI-associated tumor progression by remodeling the neural and lymphatic microenvironment. [29].

Differential expression and pathway enrichment analyses revealed distinct signaling signatures between PNI-positive and PNI-negative tumors, including the upregulation of MAPK, PI3K/AKT, and p53 pathways, alongside the downregulation of TNF and IL-17 signaling. Recent studies have shown that the MAPK pathway promotes the occurrence of tumor PNI by activating downstream transcription factors to upregulate the expression of invasion-related molecules such as matrix metalloproteinases (e.g., MMP9) for degrading the perineural matrix, responding to factors secreted by the neural microenvironment (e.g., GDNF, CCL2) to enhance the chemotaxis of tumor cells towards nerves, and promoting the expression of adhesion molecules (e.g., integrins) between tumor cells and nerve cells to strengthen their interaction [30,31,32]. The PI3K/AKT pathway promotes the occurrence of tumor PNI by activating downstream effectors (e.g., mTOR, NF-κB) to upregulate the expression of matrix metalloproteinases (e.g., MMP9) and adhesion molecules for degrading the perineural matrix, responding to signaling molecules in the neural microenvironment (e.g., CCL26, bFGF) to enhance the chemotaxis and invasion of tumor cells towards nerves, and promoting the interaction between tumor cells and nerve cells (e.g., through L1CAM, DKK1) [33,34,35]. The TNF pathway regulates the occurrence of tumor PNI through the binding of TNF-α to TNFR1/2 receptors, activating downstream molecules such as NF-κB, modulating the release of inflammatory factors like IL-6 to reshape the neural microenvironment. The pathway also regulates the expression of molecules such as MMP9, which is involved in degrading the perineural matrix and influencing tumor cell motility. Additionally, it mediates nerve–tumor interaction signals, affecting the chemotaxis of tumor cells toward nerves [36,37,38].

**Figure 5:**
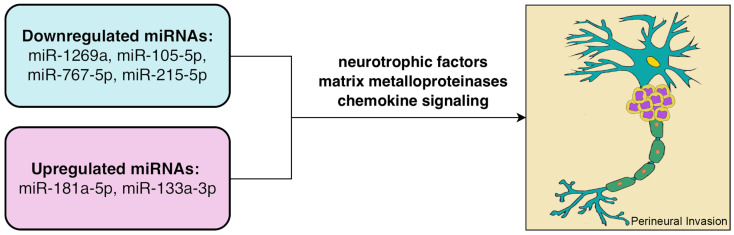
Schematic illustration of miRNA-mediated mechanisms in PNI. PNI: Perineural Invasion.

miRNAs are single-stranded non-coding RNAs of 18–25 nucleotides that regulate gene expression through RNA-induced silencing complexes. In this study, six differentially expressed miRNAs were identified between PNI-positive and PNI-negative samples. Among them, miR-1269a was significantly downregulated in the PNI-positive group and was associated with better DSS and PFI. Previous studies have shown that these miRNAs regulate tumor behavior through multiple signaling pathways and exert context-dependent functions across different cancer types; for example, miR-215 and miR-181a have been reported to promote cervical cancer progression [39,40]. miR-1269a has been shown to promote gastric cancer cell proliferation and cell cycle progression by activating the PI3K/AKT pathway via suppression of RASSF9 [41]. miR-1269a overexpression has been reported to form a positive feedback loop with TGF-β signaling, promoting epithelial–mesenchymal transition and enhancing invasion and metastasis in colorectal cancer, where it is associated with recurrence and poor prognosis [42]. miR-133a-3p is markedly downregulated in both esophageal squamous cell carcinoma and prostate cancer and exerts tumor-suppressive effects through distinct downstream targets. In esophageal squamous cell carcinoma, it directly targets COL1A1 to inhibit cell cycle progression, proliferation, and invasion while promoting apoptosis [43]. In prostate cancer, it suppresses PI3K/AKT signaling by targeting EGFR, FGFR1, IGF1R, and MET, thereby reducing cancer stem-like phenotypes, anoikis resistance, and bone metastasis [44]. However, the pathway enrichment results did not align with alterations expected from any single miRNA, indicating that PNI-associated signaling changes likely reflect coordinated regulation by multiple molecular factors. A schematic illustration of the potential mechanisms linking dysregulated miRNAs to PNI is presented in Figure 5.

Previous studies have identified several miRNAs associated with PNI, which regulate neural remodeling, tumor invasion, and pathway activation by modulating neurotrophic factors, matrix metalloproteinases, and chemokine signaling axes, thereby influencing PI3K/AKT and NF-κB signaling pathways. In addition, miRNAs can be transferred via extracellular vesicles between tumor cells and stromal components, shaping the tumor microenvironment and facilitating perineural invasion. Neural microenvironmental factors or alternative targets such as PTEN may also contribute to pathway activation [45]. Given that PNI involves multiple signaling pathways and tumor-specific biological contexts, the roles of individual miRNAs may vary across cancer types. Overall, these findings suggest that miRNA dysregulation contributes to PNI through complex regulatory networks, and further experimental validation is required to clarify the underlying mechanisms.

A major strength of this study is the relatively large cohort of surgically treated early-stage cervical cancer patients, with standardized clinical endpoints and centralized pathology review by gynecologic pathologists. S100 immunohistochemistry was applied in equivocal cases to support the assessment of PNI, enhancing diagnostic reliability. Another strength is the integration of clinical outcome data with an independent exploratory multi-omics analysis based on TCGA, which adds biological plausibility and generates hypotheses regarding tumor–nerve–immune interactions.

However, several limitations should be acknowledged. The overall recurrence rate was low at 5.4 percent (27 of 499), and the primary novel observation regarding PNI was derived from a low-risk subgroup with few events. Therefore, the findings may be most applicable to this specific subgroup and should be interpreted cautiously to avoid potential overfitting or overestimation of predictive performance. In addition, due to sample size constraints, further stratified analyses, including those based on postoperative treatment modalities, were not feasible. Larger, multi-center cohorts with longer follow-up are needed to validate these findings and refine risk stratification. The TCGA-based analysis provides a descriptive overview of molecular features associated with PNI. Grouping was based on pathological slide evaluation and may be influenced by tissue sampling location and sectioning techniques. Moreover, the identified molecular alterations have not been functionally validated and therefore we cannot establish causal relationships. While these findings offer preliminary biological context and generate hypotheses, additional experimental studies are required to confirm their mechanistic relevance.

Consistent with this exploratory framework, our multi-omics analyses suggest that PNI in cervical cancer may reflect coordinated alterations in tumor signaling, immune infiltration, and post-transcriptional regulation. These observations support the concept that tumor–nerve–immune interactions may contribute to PNI but should be regarded as hypothesis-generating and require further functional investigation. Future research should explore the integration of histotype-specific factors with PNI and Sedlis criteria to further refine personalized treatment strategies for early-stage cervical cancer.

## 5. Conclusions

Perineural invasion (PNI) was identified in 11.6% of early-stage cervical cancers and was associated with adverse pathological features, including larger tumor size, deep stromal invasion, vaginal involvement, and LVSI. While PNI was not an independent prognostic factor in the overall cohort, it was associated with an increased risk of recurrence in the subgroup of patients who did not meet Sedlis criteria, suggesting a potential role for PNI in refining risk assessment within an otherwise low-risk postoperative population. Integrating PNI into Sedlis-based prediction models yielded a modest improvement in 5-year recurrence discrimination; however, these findings are driven by a small subgroup with few events and require confirmation in independent cohorts and with appropriate model validation. Exploratory analyses in the TCGA cohort indicated PNI-associated transcriptomic and immune microenvironment differences, including an increased estimated abundance of resting mast cells, and identified PNI-associated miRNAs, with hsa-miR-1269a downregulation associated with PNI and survival. Overall, these results support PNI as a marker of aggressive tumor biology and generate hypotheses regarding tumor–nerve–immune interactions, warranting further validation and mechanistic studies before clinical implementation.

## Figures and Tables

**Table 1:** Demographic and clinicopathologic features stratified by PNI.

Characteristics		PNI		*p*-Value
		Negative (*n* = 441)	Positive (*n* = 58)	
Age, years	<60	400 (90.7)	52 (89.7)	0.797
	≥60	41 (9.3)	6 (10.3)	
Conization	no	402 (91.2)	55 (94.8)	0.455
	yes	39 (8.8)	3 (5.2)	
FIGO stage	IB	369 (83.7)	49 (84.5)	0.875
	IIA	72 (16.3)	9 (15.5)	
Tumor size, cm	≤2	160 (36.3)	10 (17.2)	0.002
	2~4	206 (46.7)	29 (50.0)	
	>4	75 (17.0)	19 (32.8)	
Histology	SCC	373 (84.6)	47 (81.0)	0.487
	AC/ASC	68 (15.4)	11 (19.0)	
Grade	G1 and G2	426 (96.6)	54 (93.1)	0.260
	G3	15 (3.4)	4 (6.9)	
Vaginal invasion	negative	350 (79.4)	38 (65.5)	0.017
	positive	91 (20.6)	20 (34.5)	
Depth of invasion	superficial 1/3	171 (38.8)	9 (15.5)	<0.001
	middle 1/3	154 (34.9)	17 (29.3)	
	deep 1/3	116 (26.3)	32 (55.2)	
LVSI	negative	244 (55.3)	24 (41.4)	0.045
	positive	197 (44.7)	34 (58.6)	
LNM	negative	386 (87.5)	48 (82.8)	0.310
	positive	55 (12.5)	10 (17.2)	
5-year RFS	alive	405 (91.8)	50 (86.2)	0.155
	dead	36 (8.2)	8 (13.8)	
5-year OS	alive	426 (96.6)	54 (93.1)	0.260
	dead	15 (3.4)	4 (6.9)	
Follow-up time, months		54.29 (46.00–63.00)	52.62 (44.75–61.25)	

Note: *p* < 0.05 is considered statistically significant. Abbreviations: FIGO: The International Federation of Gynecology and Obstetrics; LVSI: Lymphovascular Invasion; LNM: Lymph Node Metastasis; RFS: Recurrence-Free Survival; OS: Overall Survival.

**Table 2:** Univariate analysis for 5-year RFS and 5-year OS.

Characteristics		5-Year RFS	*p*-Value	5-Year OS	*p*-Value
		Univariate Analysis OR (95% CI)		Univariate Analysis OR (95% CI)	
Age, years	<60	1	0.124	1	0.534
	≥60	0.207 (0.028–1.537)		0.524 (0.068–4.017)	
Conization	no	1	0.866	1	0.736
	yes	1.097 (0.373–3.232)		1.294 (0.289–5.803)	
FIGO stage	IB	1	0.103	1	0.232
	IIA	1.833 (0.885–3.798)		1.898 (0.664–5.426)	
Tumor size, cm	≤2	1	0.018	1	0.003
	2~4	1.664 (0.738–3.751)	0.219	5.956 (0.738–48.075)	0.094
	>4	3.397 (1.424–8.101)	0.006	20.119 (2.533–159.793)	0.005
Histology	SCC	1	0.193	1	0.210
	AC/ASC	1.645 (0.777–3.484)		1.959 (0.685–5.604)	
Grade	G1 and G2	1	0.582	1	0.737
	G3	0.565 (0.074–4.333)		1.426 (0.180–11.278)	
Vaginal invasion	negative	1	0.225	1	0.126
	positive	1.5228 (0.770–3.031)		2.109 (0.810–5.492)	
Depth of invasion	superficial 1/3	1	0.010	1	0.205
	middle 1/3	1.283 (0.539–3.052)	0.573	1.600 (0.444–5.771)	0.473
	deep 1/3	1.968 (1.358–6.489)	0.006	2.849 (0.859–9.446)	0.087
LVSI	negative	1	0.001	1	0.021
	positive	3.040 (1.550–5.963)		3.394 (1.203–9.571)	
PNI	negative	1	0.160	1	0.201
	positive	1.800 (0.792–4.089)		2.104 (0.674–6.569)	
LNM	negative	1	<0.001	1	0.004
	positive	4.735 (2.394–9.365)		4.244 (1.606–11.215)	
Sedlis criteria	low-risk	1	<0.001	1	0.003
	high-risk	3.711 (1.890–7.284)		5.528 (1.808–16.908)	

Note: *p* < 0.05 is considered statistically significant. Abbreviations: RFS: Regression-Free Survival; OS: Overall Survival; OR: Odds Ratio; CI: Confidence Interval; FIGO: The International Federation of Gynecology and Obstetrics; LVSI: Lymphovascular Invasion; PNI: Perineural Invasion; LNM: Lymph Node Metastasis.

**Table 3:** Multivariate analysis for 5-year RFS and 5-year OS.

Characteristics		5-Year RFS	*p*-Value	5-Year OS	*p*-Value
		Multivariate Analysis OR (95% CI)		Multivariate Analysis OR (95% CI)	
Tumor size, cm	≤2	Reference	0.567	Reference	0.021
	2~4	1.290 (0.544,3.060)	0.564	5.123 (0.611,42.931)	0.132
	>4	1.714 (0.631,4.652)	0.290	14.812 (1.698,129.218)	0.015
Depth of invasion	superficial 1/3	Reference	0.639	Reference	0.865
	middle 1/3	0.924 (0.369,2.312)	0.866	0.932 (0.244,3.554)	0.918
	deep 1/3	1.350 (0.540,3.378)	0.521	0.713 (0.181,2.812)	0.628
LVSI	negative	Reference		Reference	
	positive	2.204 (1.071,4.534)	0.032	2.383 (0.792,7.175)	0.122
PNI	negative	Reference		Reference	
	positive	1.261 (0.519,3.063)	0.608	1.366 (0.407,4.587)	0.613
LNM	negative	Reference		Reference	
	positive	3.176 (1.505,6.700)	0.002	2.391 (0.797,7.175)	0.120

Note: *p* < 0.05 is considered statistically significant. Abbreviations: RFS: Regression-Free Survival; OS: Overall Survival; OR: Odds Ratio; CI: Confidence Interval; LVSI: Lymphovascular Invasion; PNI: Perineural Invasion; LNM: Lymph Node Metastasis.

## Data Availability

The data presented in this study are available on request from the corresponding author due to our institution’s patient data management policies.

## Abbreviations

The following abbreviations are used in this manuscript:

PNI	Perineural Invasion
LVSI	Lymphovascular Invasion
RFS	Recurrence-Free Survival
OS	Overall Survival
CESC	Cervical Squamous Cell Carcinoma
FIGO	The International Federation of Gynecology and Obstetrics
LNM	Lymph Node Metastasis
OR	Odds Ratio
CI	Confidence Interval
DEGs	Differentially Expressed Genes

## References

[1] Siegel R.L., Miller K.D., Wagle N.S., Jemal A. (2023). Cancer statistics, 2023. CA Cancer J. Clin..

[2] Sedlis A., Bundy B.N., Rotman M.Z., Lentz S.S., Muderspach L.I., Zaino R.J. (1999). A randomized trial of pelvic radiation therapy versus no further therapy in selected patients with stage IB carcinoma of the cervix after radical hysterectomy and pelvic lymphadenectomy: A Gynecologic Oncology Group Study. Gynecol. Oncol..

[3] Liebig C., Ayala G., Wilks J.A., Berger D.H., Albo D. (2009). Perineural invasion in cancer: A review of the literature. Cancer.

[4] Takahashi H., Ohigashi H., Ishikawa O., Gotoh K., Yamada T., Nagata S., Tomita Y., Eguchi H., Doki Y., Yano M. (2012). Perineural invasion and lymph node involvement as indicators of surgical outcome and pattern of recurrence in the setting of preoperative gemcitabine-based chemoradiation therapy for resectable pancreatic cancer. Ann. Surg..

[5] Ozcan F. (2001). Correlation of perineural invasion on radical prostatectomy specimens with other pathologic prognostic factors and PSA failure. Eur. Urol..

[6] Shen F.Z., Zhang B.Y., Feng Y.J., Jia Z.X., An B., Liu C.C., Deng X.Y., Kulkarni A.D., Lu Y. (2010). Current research in perineural invasion of cholangiocarcinoma. J. Exp. Clin. Cancer Res..

[7] Shimada K., Nara S., Esaki M., Sakamoto Y., Kosuge T., Hiraoka N. (2011). Intrapancreatic nerve invasion as a predictor for recurrence after pancreaticoduodenectomy in patients with invasive ductal carcinoma of the pancreas. Pancreas.

[8] Zhang F., Chen H., Luo D., Xiong Z., Li X., Yin S., Jin L., Chen S., Peng J., Lian L. (2023). Lymphovascular or perineural invasion is associated with lymph node metastasis and survival outcomes in patients with gastric cancer. Cancer Med..

[9] Cai G., Zhang S., Gao S., Deng T., Huang H., Feng Y., Wan T. (2025). What is the impact of perineural invasion on the prognosis of cervical cancer: A systematic review and meta-analysis. BMC Cancer.

[10] Memarzadeh S., Natarajan S., Dandade D.P., Ostrzega N., Saber P.A., Busuttil A., Lentz S.E., Berek J.S. (2003). Lymphovascular and perineural invasion in the parametria: A prognostic factor for early-stage cervical cancer. Obstet. Gynecol..

[11] Horn L.C., Meinel A., Fischer U., Bilek K., Hentschel B. (2010). Perineural invasion in carcinoma of the cervix uteri--prognostic impact. J. Cancer Res. Clin. Oncol..

[12] Long Y., Yao D.S., Wei Y.S., Wu G.T. (2018). Effects of Nerve Growth Factor Expression on Perineural Invasion and Worse Prognosis in Early-Stage Cervical Cancer. Chin. Med. J..

[13] Zhang G., Yang Y., Zhu Y., Cui L., Jia S., Shi Y., Song S., Xu S. (2015). Evidence of perineural invasion on early-stage cervical cancer and prognostic significance. Zhonghua Fu Chan Ke Za Zhi.

[14] Wan T., Tu H., Liu L., Huang H., Feng Y., Liu J. (2021). Perineural Invasion Should Be Regarded as an Intermediate-Risk Factor for Recurrence in Surgically Treated Cervical Cancer: A Propensity Score Matching Study. Dis. Markers.

[15] Fernandez-Nogueira P., Linzoain-Agos P., Cueto-Remacha M., De la Guia-Lopez I., Recalde-Percaz L., Parcerisas A., Gascon P., Carbo N., Gutierrez-Uzquiza A., Fuster G. (2024). Role of semaphorins, neuropilins and plexins in cancer progression. Cancer Lett..

[16] Silverman D.A., Martinez V.K., Dougherty P.M., Myers J.N., Calin G.A., Amit M. (2021). Cancer-Associated Neurogenesis and Nerve-Cancer Cross-talk. Cancer Res..

[17] Zhu Z., Zhang X., Guo H., Fu L., Pan G., Sun Y. (2015). CXCL13-CXCR5 axis promotes the growth and invasion of colon cancer cells via PI3K/AKT pathway. Mol. Cell. Biochem..

[18] Xia Y., Jiang T., Li Y., Gu C., Lv J., Lu C., Xu P., Fang L., Chen Z., Liu H. (2024). circVAPA-rich small extracellular vesicles derived from gastric cancer promote neural invasion by inhibiting SLIT2 expression in neuronal cells. Cancer Lett..

[19] Baraldi J.H., Martyn G.V., Shurin G.V., Shurin M.R. (2022). Tumor Innervation: History, Methodologies, and Significance. Cancers.

[20] Zhang M., Xin L., Cheng B., Yan B., Zhen J., Yang C., Ma L., Hou Q. (2023). Clinical Value Analysis of Serum TK1, SCC-Ag, and MUC-1 in the Diagnosis and Prognosis Evaluation of Cervical Cancer. Altern. Ther. Health Med..

[21] Ratan C., Cicily K.D.D., Nair B., Nath L.R. (2021). MUC Glycoproteins: Potential Biomarkers and Molecular Targets for Cancer Therapy. Curr. Cancer Drug Targets.

[22] Mancusi de Carvalho J.P., Salim R.C., Carvalho F.M., Nogueira Dias Genta M.L., Baracat E.C., Carvalho J.P. (2020). L1 cell adhesion molecule (L1CAM) in stage IB cervical cancer: Distinct expression in squamous cell carcinomas and adenocarcinomas. J. Clin. Pathol..

[23] Benbrook D.M., Deng W., Gold M.A., Rai R., Conrad R., van der Wel H., Husain S., Moore K., Spirtos N., Jackson A.L. (2023). Association of Sialyl Tn antigen with cervical cancer lymph node status: An NRG oncology/GOG study. Gynecol. Oncol..

[24] Gao X., Wang Q., Huang T., Xu C., Yang X., Zhang L., Wang J., Yang L., Zheng X., Fan Q. (2024). Cervical cancer-produced neuromedin-B reprograms Schwann cells to initiate perineural invasion. Cell Death Dis..

[25] Chen G., Zheng Z., Sun H., You J., Chu J., Gao J., Qiu L., Liu X. (2023). Dedifferentiated Schwann cells promote perineural invasion mediated by the PACAP paracrine signalling in cervical cancer. J. Cell Mol. Med..

[26] Wan T., Deng T., Peng X., Cai G., Huang H., Ling Y., Gao S., Li H., Yu D., Li H. (2025). Comprehensive Multi-Omic Characterization of Perineural Invasion in Cervical Cancer Reveals Diagnostic Markers, Molecular Drivers, and Therapeutic Strategies. Cancer Res..

[27] Liu J., Zhang Y., Zhao J., Yang Z., Li D., Katirai F., Huang B. (2011). Mast cell: Insight into remodeling a tumor microenvironment. Cancer Metastasis Rev..

[28] Forsythe P. (2019). Mast Cells in Neuroimmune Interactions. Trends Neurosci..

[29] Keser S.H., Kandemir N.O., Ece D., Gecmen G.G., Gul A.E., Barisik N.O., Sensu S., Buyukuysal C., Barut F. (2017). Relationship of mast cell density with lymphangiogenesis and prognostic parameters in breast carcinoma. Kaohsiung J. Med. Sci..

[30] He S., Chen C.H., Chernichenko N., He S., Bakst R.L., Barajas F., Deborde S., Allen P.J., Vakiani E., Yu Z. (2014). GFRalpha1 released by nerves enhances cancer cell perineural invasion through GDNF-RET signaling. Proc. Natl. Acad. Sci. USA.

[31] He S., He S., Chen C.H., Deborde S., Bakst R.L., Chernichenko N., McNamara W.F., Lee S.Y., Barajas F., Yu Z. (2015). The chemokine (CCL2-CCR2) signaling axis mediates perineural invasion. Mol. Cancer Res..

[32] Chen X., An Y., Zhang Y., Xu D., Chen T., Yang Y., Chen W., Wu D., Zhang X. (2021). CCL26 is upregulated by nab-paclitaxel in pancreatic cancer-associated fibroblasts and promotes PDAC invasiveness through activation of the PI3K/AKT/mTOR pathway. Acta Biochim. Biophys. Sin..

[33] Wang J., Li Q., Liang F., Du X., Song P., Wu T., Chen R., Lin X., Liu Q., Hu H. (2024). Dickkopf-1 drives perineural invasion via PI3K-AKT signaling pathway in head and neck squamous cancer. MedComm.

[34] Kim J.H., Lee K.W., Ahn D.G., Oh K.Y., Yoon H.J. (2023). Clinical significance of L1CAM expression and its biological role in the progression of oral squamous cell carcinoma. Oncol. Rep..

[35] Hansen F.J., David P., Akram M., Knoedler S., Mittelstadt A., Merkel S., Podolska M.J., Swierzy I., Rossdeutsch L., Klosch B. (2023). Circulating Monocytes Serve as Novel Prognostic Biomarker in Pancreatic Ductal Adenocarcinoma Patients. Cancers.

[36] Bostanghadiri N., Razavi S., Shariati A., Talebi M., Mirkalantari S., Emami Razavi A., Darban-Sarokhalil D. (2023). Exploring the interplay between Fusobacterium nucleatum with the expression of microRNA, and inflammatory mediators in colorectal cancer. Front. Microbiol..

[37] Zhou Y., Qin Y., Ma J., Li Z., Heng W., Zhang L., Liu H., Li R., Zhang M., Peng Q. (2024). Heat-killed Prevotella intermedia promotes the progression of oral squamous cell carcinoma by inhibiting the expression of tumor suppressors and affecting the tumor microenvironment. Exp. Hematol. Oncol..

[38] Kori M., Yalcin Arga K. (2018). Potential biomarkers and therapeutic targets in cervical cancer: Insights from the meta-analysis of transcriptomics data within network biomedicine perspective. PLoS ONE.

[39] Xie Z., Zhong C., Duan S. (2022). miR-1269a and miR-1269b: Emerging Carcinogenic Genes of the miR-1269 Family. Front. Cell Dev. Biol..

[40] Xu H., Zhu J., Hu C., Song H., Li Y. (2016). Inhibition of microRNA-181a may suppress proliferation and invasion and promote apoptosis of cervical cancer cells through the PTEN/Akt/FOXO1 pathway. J. Physiol. Biochem..

[41] Liu W.L., Wang H.X., Shi C.X., Shi F.Y., Zhao L.Y., Zhao W., Wang G.H. (2019). MicroRNA-1269 promotes cell proliferation via the AKT signaling pathway by targeting RASSF9 in human gastric cancer. Cancer Cell. Int..

[42] Bu P., Wang L., Chen K.Y., Rakhilin N., Sun J., Closa A., Tung K.L., King S., Kristine Varanko A., Xu Y. (2015). miR-1269 promotes metastasis and forms a positive feedback loop with TGF-beta. Nat. Commun..

[43] Yin Y., Du L., Li X., Zhang X., Gao Y. (2019). miR-133a-3p suppresses cell proliferation, migration, and invasion and promotes apoptosis in esophageal squamous cell carcinoma. J. Cell. Physiol..

[44] Tang Y., Pan J., Huang S., Peng X., Zou X., Luo Y., Ren D., Zhang X., Li R., He P. (2018). Downregulation of miR-133a-3p promotes prostate cancer bone metastasis via activating PI3K/AKT signaling. J. Exp. Clin. Cancer Res..

[45] Zhang M., Xian H.C., Dai L., Tang Y.L., Liang X.H. (2021). MicroRNAs: Emerging driver of cancer perineural invasion. Cell. Biosci..

